# The Patterns and Puzzles of Genetic Diversity of Endangered Freshwater Mussel *Unio crassus* Philipsson, 1788 Populations from Vistula and Neman Drainages (Eastern Central Europe)

**DOI:** 10.3390/life10070119

**Published:** 2020-07-21

**Authors:** Adrianna Kilikowska, Monika Mioduchowska, Anna Wysocka, Agnieszka Kaczmarczyk-Ziemba, Joanna Rychlińska, Katarzyna Zając, Tadeusz Zając, Povilas Ivinskis, Jerzy Sell

**Affiliations:** 1 Department of Genetics and Biosystematics, Faculty of Biology, University of Gdańsk, Wita Stwosza 59, 80-308 Gdańsk, Poland; adrianna.kilikowska@biol.ug.edu.pl (A.K.); monika.mioduchowska@ug.edu.pl (M.M.); anna.wysocka@ug.edu.pl (A.W.); agnieszka.kaczmarczyk-ziemba@ug.edu.pl (A.K.-Z.); joannry@gmail.com (J.R.); jerzy.sell@biol.ug.edu.pl (J.S.); 2 Department of Marine Plankton Research, University of Gdansk, Piłsudskiego 46, 81-378 Gdynia, Poland; 3 Institute of Nature Conservation, Polish Academy of Sciences, 31-120 Kraków, Poland; tzajac@iop.krakow.pl; 4 Nature Research Centre, Akademijos 2, LT-08412 Vilnius, Lithuania; ivinskis@ekoi.lt

**Keywords:** *Unio crassus*, freshwater mussels, population genetics, genetic diversity, mtDNA, ITS

## Abstract

Mussels of the family Unionidae are important components of freshwater ecosystems. Alarmingly, the International Union for Conservation of Nature and Natural Resources Red List of Threatened Species identifies almost 200 unionid species as extinct, endangered, or threatened. Their decline is the result of human impact on freshwater habitats, and the decrease of host fish populations. The Thick Shelled River Mussel *Unio crassus* Philipsson, 1788 is one of the examples that has been reported to show a dramatic decline of populations. Hierarchical organization of riverine systems is supposed to reflect the genetic structure of populations inhabiting them. The main goal of this study was an assessment of the *U. crassus* genetic diversity in river ecosystems using hierarchical analysis. Different molecular markers, the nuclear ribosomal internal transcribed spacer ITS region, and mitochondrial DNA genes (*cox1* and *ndh1*), were used to examine the distribution of *U. crassus* among-population genetic variation at multiple spatial scales (within rivers, among rivers within drainages, and between drainages of the Neman and Vistula rivers). We found high genetic structure between both drainages suggesting that in the case of the analyzed *U. crassus* populations we were dealing with at least two different genetic units. Only about 4% of the mtDNA variation was due to differences among populations within drainages. However, comparison of population differentiation within drainages for mtDNA also showed some genetic structure among populations within the Vistula drainage. Only one haplotype was shared among all Polish populations whereas the remainder were unique for each population despite the hydrological connection. Interestingly, some haplotypes were present in both drainages. In the case of *U. crassus* populations under study, the Mantel test revealed a relatively strong relationship between genetic and geographical distances. However, in detail, the pattern of genetic diversity seems to be much more complicated. Therefore, we suggest that the observed pattern of *U. crassus* genetic diversity distribution is shaped by both historical and current factors i.e. different routes of post glacial colonization and history of drainage systems, historical gene flow, and more recent habitat fragmentation due to anthropogenic factors.

## 1. Introduction

Mussels of the family Unionidae with 680 described species [1] are important components of freshwater ecosystems. Alarmingly, the International Union for Conservation of Nature and Natural Resources Red List identifies almost 200 species of this family as extinct, endangered, or threatened [2] which makes unionids one of the most endangered groups of invertebrates in the world [3,4]. Their decline is the result of ever-increasing human impact on freshwater habitats, such as the regulation and impoundment of rivers or water pollution. The decline of host fish populations also has an effect on extirpation of freshwater mussels as their larvae (glochidia) are parasites on fish, which serve to complete their life cycle and disperse progeny [3,5,6,7,8,9].

In Europe, the remarkable decline suffered by freshwater mussel populations has attracted the attention of conservation organizations [10]. Over recent years some comprehensive studies have been published concerning biology, ecology, phylogeny, and conservation status of European freshwater mussels (e.g., [11,12,13,14,15,16,17,18,19,20,21,22,23]). This is all the more important in that the situation of freshwater bivalves in Europe is even more alarming than in North America. Among European freshwater mussel species, 75% of the species are categorized as Threatened or near Threatened [21]. In comparison with other taxa in Europe (e.g., fish, amphibians, birds, mammals), the real situation is poorly understood and especially alarming due to much lower species biodiversity within the whole superfamily Unionoidea. 

Much of the global awareness of freshwater mussel decline stems from North American Unionoidea, which constitutes the continent’s most imperiled fauna, but much more numerous in terms of species than the rest of the continents [24,25]. Over 70% of North American species are considered imperiled at some level [24] and more than 25 species are presumed extinct (see [4,26] for details). Consequently, many more studies have been focused on Unionoidea species from North America and many of these have reported upon molecular based phylogenies of this group of mollusks (e.g., [27,28,29,30,31,32,33,34,35,36]).

The Thick Shelled River Mussel, *Unio crassus* Philipsson, 1788 is one of the examples that has been reported to show a dramatic decline of populations within the western and central European part of its range and thus has become a major target species for conservation [37,38,39,40]. In the eastern part of the mussel range the situation is better [41]. Up to the 20th century *U. crassus* occupied and colonized a wide range of habitats and was considered the most abundant unionid species in central and northern Europe ([42] and references therein). In Poland, up to the last century it had been also a dominant species in many rivers, reaching extremely high densities [43]. However, today its numbers have fallen dramatically, in line with the deterioration of water quality. Although potentially harmful effects of anthropogenic activities, including water pollution and flow modification, have been reduced in central Europe over the last decades, *U. crassus* populations are not recovering accordingly ([44] and references therein). Consequently, it has been listed as endangered (category EN) in the IUCN Red List of threatened species [2] as well as protected in Europe e.g., in the European Union by the enclosed Appendixes II and IV of the Habitats Directive. 

Preservation of biodiversity requires not only the protection of individual taxa but also the preservation of genetic diversity. Although molecular data seem to be critical for the conservation management of imperiled freshwater mussels, the knowledge about genetic diversity of *U. crassus* populations is still very scarce. It seems that first Nagel and Badino (2001) [45] reported on the genetic variability within and between *U. crassus* populations. However, the basic aim of that study was to solve the taxonomic and phylogenetic problems within Unionidae. Moreover, the study was based on allozyme markers, which are widely known to underestimate genetic variation, especially on the recent time scale and to be influenced by natural selection. 

Since then, other molecular investigations including a limited number of *U. crassus* individuals have been reported. However, these studies analyzed taxonomic uncertainties at the genus or family level, establish phylogenetic relationships, biogeographic patterns or evolutionary history of European unionids rather than focusing on population genetics of this species (e.g., [21,46,47,48]). More precisely, Prié and Puillandre (2014) [49] used mitochondrial DNA (mtDNA) data of *Unio* species from France (including limited samples of *U. crassus*) to clarify the unionid taxonomy in this country. Similar taxonomic studies have been performed in the area covering Russia and Ukraine [50,51]. Except for analyses based on mtDNA, there have also been investigations of the phylogenetic relationships within unionids using sequences of nuclear DNA (nDNA) and the transcribed spacers ITS1 and ITS2 [52]. Some *U. crassus* sequences have been used to evaluate an application of the ITS region as a phylogenetic marker [12]. Using the distribution data and multi-locus phylogeny (COI, 16S rRNA, 28S rRNA) Bolotov et al. (2020) [41] described the actual taxonomic richness of Unionidae in Russia and Kazakhstan with distribution patterns for each genus and species including *U. crassus*. Feind et al. (2018) [53] reported results of the *U. crassus* populations structure analyses in six major drainage systems in Germany and Sweden using a set of nine microsatellite markers. Additionally, mitochondrial sequences of *U. crassus* have been used to establish the utility of paternally-inherited markers in phylogeographical studies [54]. 

Thus, given the extremely limited genetic data on populations of *U. crassus*, studies of the genetic diversity and population structure of this species are needed. Especially that, recent analyses of another endangered freshwater mussel—*Margaritifera margaritifera* (Linnaeus, 1758)—have demonstrated that knowledge of the genetic structure of populations can be extremely useful for their conservation [3,5,6,55,56,57].

In general, genetic variability of a species is partitioned into variation within and among populations and groups of populations. The opposing forces of genetic drift and gene flow determine the relative proportion of neutral genetic variation within each of these two components [58]. Genetic drift results in loss of genetic diversity within a population and at the same time promotes differentiation of populations [59]. Conversely, gene flow among populations tends to increase variation within populations, while minimizing differences among these populations. In aquatic invertebrates, dispersal is a major component of gene flow among populations [60]. The effects of genetic drift occur more rapidly in small populations, and isolation promotes population differentiation [61]. Reduction of a within-population variation due to genetic drift increases the probability of extinction [62,63,64]. In addition, genetic changes due to isolation will increase the genetic structure among populations. Thus, preservation of the genetic diversity within a conservation framework requires understanding of both within-population genetic variation and patterns of variation among populations across the species geographical range landscape. Such knowledge seems to be extremely important in the case of endangered freshwater mussels.

Because of the hierarchical organization of riverine systems (first order streams giving rise to second order streams, etc.), populations of organisms within these systems are likely to have a genetic structure that reflects such a type of organization. Thus, hierarchical analysis of genetic variation is a particularly useful approach for genetic diversity assessment of riverine organisms ([32] and references therein). 

In the present study, partial DNA sequence data of two mitochondrial genes—NADH dehydrogenase subunit 1 (*ndh1*) and cytochrome oxidase subunit I (*cox1*) as well as the entire ITS region of the nuclear ribosomal DNA (herein called nrDNA)—were used to:

(i) test the correlation between the genetic differentiation and geographic isolation of *U. crassus* populations using a hierarchical approach,
(ii) examine the distribution of genetic variation among populations of *U. crassus* at multiple spatial scales (within rivers, among rivers within drainages, and across drainages),
(iii) test the role of current vs historical gene flow into the distribution of *U. crassus* genetic diversity on the basis of populations representing currently isolated Neman and Vistula drainages (Central Europe) with the reported ancient connection,
(iv) asses the phylogenetic affinities among *U. crassus* populations.

## 2. Materials and Methods

### 2.1. Sample Collection and Identification

**Figure 1:**
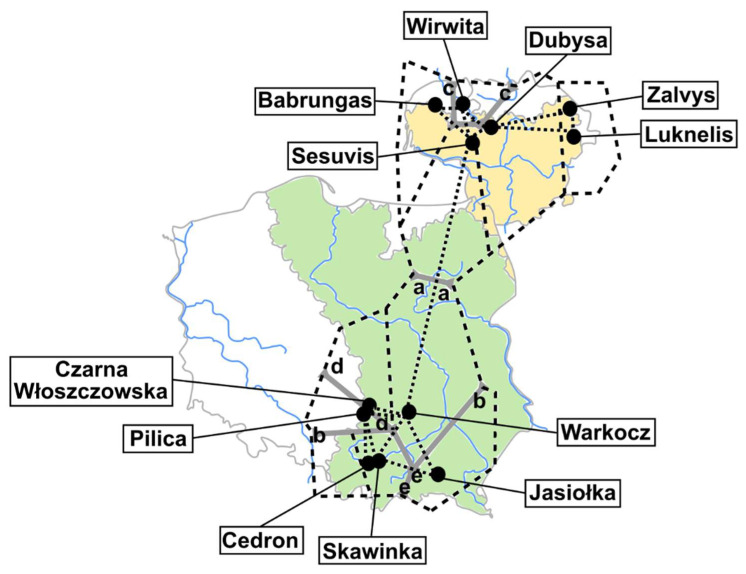
Sampling sites and spatial analysis based on Monmonier’s maximum difference algorithm [65] for detecting genetic barriers (genetic breaks) among populations of *U. crassus*. Vistula and Neman drainages are marked with different colors, identified barriers are indicated as bold grey lines (the first five barriers are shown, from “a” to “e”), the Delaunay triangulation is visualized as black dotted lines. Locality codes: CED–Cedron, CZW–Czarna Włoszczowska, JAS–Jasiołka, PIL–Pilica, SKA–Skawinka, WAR–Warkocz, BAB–Babrungas, DUB–Dubysa, LUK–Luknelis, SES–Sesuvis, VIR–Virvita, ZAL–Zalvys.

The conservation status of *U. crassus* categorized as Endangered species prevented us from dealing with large numbers of specimens, which were sometimes below the recommended figures for population structure analyses. Nevertheless, this is currently an insurmountable problem that should not dissuade us from obtaining as much data as possible on these endangered species. In total, 99 specimens representing *U. crassus* species were collected from 6 localities in Poland and 6 in Lithuania (Figure 1, Tables S1 and S2). Moreover, individuals of *Unio tumidus* Philipsson, 1788 were collected from the Pilica river and used as an outgroup in the analyses.

Samples of *U. crassus* were collected from habitats localized in central and southern Poland, from EU protected areas of the Natura 2000. Additional samples were also collected from selected rivers in Lithuania, also within the Natura 2000 areas. Details on sampling localities are presented in Table S2. The available reports on the monitoring results indicate that overall conservation status of the *U. crassus* is unsatisfactory—assessments of conservation status of the species under Article 17 of the Habitats Directive in period 2013–2018 for Poland, Lithuania and EU are available at “Article 17 web tool” [66].

In the sampling design (Figure 1, Table S2) we considered the distribution of among-population genetic variation at multiple spatial scales: i) within rivers and its tributaries (the Skawinka river with its tributary the Cedron; the Pilica river with its tributary the Czarna Włoszczowska); ii) among rivers within drainages: The Vistula drainage (Skawinka, Cedron, Pilica, Czarna Włoszczowska, Jasiołka, Warkocz); also having regard to division for the Upper (Cedron, Jasiołka, Skawinka, Warkocz) and the Middle Vistula (Pilica, Czarna Włoszczowska,), and the Neman drainage (Babrungas, Luknelis, Dubysa, Zalvys); Virvicia (tributary of Venta) was also included in the following analyses of Neman drainage due to the Windawski canal of only 15 km in length connecting Dubysa and Venta); iii) across drainages.

Specimens were collected and transported to the laboratory alive in water from the individual localities. Identification down to the species level was based upon morphological diagnostic characters provided by Piechocki and Dyduch-Falniowska (1993) [67]. A small fragment of somatic tissue (gills) was taken from each individual and then immediately frozen at −80 °C. Individuals from Lithuania were preserved in 96% ethanol.

### 2.2. Extraction, PCR Amplification and Sequencing

DNA was extracted from a small piece of gill tissue of each specimen using a modified phenol/chloroform method [68]. Since only somatic tissue was used, we assumed that we had extracted only F-type mitochondria.

The entire ITS region of nrDNA was amplified using the primers ITS4 (5’– TCCTCCGCTTATTGATATGC–3’) and ITS5 (5’–GGAAGTAAAAGTCGTAACAAGG–3’) of [69], annealing to the 5’ end of 28S and 3’ end of 18S rRNA genes, respectively. Parts of the NADH dehydrogenase, subunit 1 (*ndh1*) and cytochrome c oxidase subunit 1 (*cox1*) genes from mtDNA were amplified with the primer combinations: for *ndh1* 5’–TGGCAGAAAAGTGCATCAGATTTAAGC–3’ and 5’–GCTATTAGTAGGTCGT ATCG–3’ [70,71], for *cox1* 5’–GTTCCACAAATCATAAGGATATTGG–3’ and 5’–TACACCTCAGGGTGACCAAA AAACCA–3’ [72], respectively.

All PCR reactions were performed in 25 mL volumes with 0.4 μL 5 μM of each primer and about 10 ng of template using a cycling profile of 95 °C for 5 min, followed by 30 cycles of 95 °C for 30 s, 48 °C, 50 °C, 51 °C for 60 s, and 72 °C for 60 s. The mentioned annealing temperature was used for the *cox1*, *ndh1,* and nrDNA, respectively. For some specimens it was impossible to amplify all three fragments of DNA (Table S1). PCR products were cleaned up by exonuclease I (20 U/μL, Thermo Scientific) and alkaline phosphatase FastAP (1 U/μL, Thermo Scientific) treatment according to the manufacturer’s guidelines, and sequenced directly in both directions using the same primers as at the amplification stage.

### 2.3. DNA Sequence Analysis 

To verify the identity of the amplified region, BLAST [73] searches at the National Center for Biotechnology Information NCBI were performed. Sequences were quality checked and trimmed to the same length in BioEdit version 7.2.5 [74] and a consensus sequence was created for each individual. In the case of *cox1* and *ndh1* genes, the sequences could be unambiguously aligned without inserting gaps. The sequences of nrDNA were aligned using Clustal Omega [75] with default settings. Although the nrDNA region alignment had several indels, all specimens yielded sequences that were readily readable without cloning and were therefore included in the analyses. In total, 242 new sequences of *U. crassus* were obtained and deposited in GenBank (Table S1): 83 sequences of *cox1* (614 bp-long), 94 sequences of *ndh1* (859 bp-long), and 65 sequences of the nrDNA region (879 bp-long).

Data from each of the three regions were analyzed separately. Moreover, when the sequences of both mtDNA genes (*cox1* and *ndh1*) were known for particular individuals (Table S1), the data were concatenated and analyzed as a single mtDNA locus. To analyze the mitochondrial gene pairs as a single locus, congruence among tree topologies of *cox1* and *ndh1* regions was assessed by the partition homogeneity test in PAUP*.

Two data sets were analyzed for each DNA region; one consisted of only the *U. crassus* sequences, while the second set comprised also *U. tumidus* as an outgroup (newly obtained *U. tumidus* sequences were also deposited in GenBank–Acc. Nos: KJ525923–KJ525927; KJ525965, KJ525966).

Alignment statistics and DNA polymorphism, quantified as the number of haplotypes (n), haplotypic diversity (h) and nucleotide diversity (π) were calculated in DnaSP v5.10.01 [76].

We used the Arlequin v.3.5. software [77] for analysis of molecular variation. This software takes into account both haplotype frequency and molecular sequence divergence. Genetic differentiation was analyzed within-drainage for the Vistula (VIS) and Neman (NEM) drainages. We calculated genetic variation partitioned into three hierarchical levels using analysis of molecular variance (AMOVA) [77]: within populations (Φ_ST_), between populations within rivers and its tributaries (Φ_SR_), and among rivers (Φ_RT_). We then calculated among-drainage variation using three hierarchical levels: within populations (Φ_ST_), among populations within drainages (Φ_SD_), and between drainages (Φ_DT_). The number of migrants and pairwise differentiation between populations were calculated using Φ_ST_, a direct analogue of Wright’s F_ST_ for nucleotide sequence divergence. Also, these estimates were calculated for data grouped according to drainage (VIS, NEM) and hydrological division (rivers of southern Poland representing the Upper Vistula, rivers of central Poland representing the Middle Vistula). The significance of this estimate was calculated using 10,000 permutations of the data and was considered significant at P = 0.05.

To test for isolation by distance, the shortest geographic distance between populations was calculated using the Earth great circle distances calculator [78] and F_ST_ values for population pairs were tested for correlation with distance using Mantel’s test [79] as implemented in Arlequin v.3.5.

For mtDNA, we calculated a network of all individual gene sequences using the median-joining (MJ) algorithm implemented in Network v. 4.5.1.6 software [80] The method groups related haplotypes through median vectors into a tree or network. Different settings for the homoplasy level parameter (ε) were tested, and ε = 20 was eventually used. To account for differences in substitution rates the weight of 1 for transitions and 2 for transversions was applied. Ambiguous relationships were resolved with a Maximum Parsimony (MP) heuristic algorithm. With this analysis, we could determine the relationship among haplotypes and the frequencies of these haplotypes in our sampling.

In addition, we estimated the phylogenetic relationship among *U. crassus* haplotypes with *U. tumidus* sequences as an outgroup. First, the most appropriate model of sequences evolution was determined, and the nucleotide substitution parameters were estimated by jModelTest 2 [81] using Bayesian Information Criterion. For the concatenated mtDNA data, the preferred model of nucleotide substitution was the Hasegawa, Kishino, Yano 85 (HKY) [82] model. The likelihood-estimated transition/transversion ratio was 2.211. In the case of the nrDNA region, the selected model was the Kimura’s two-parameter model (K2P) [83] with gamma-distributed rate heterogeneity (Γ = 0.512). The likelihood-estimated transition/transversion ratio was 0.882. Maximum likelihood trees were calculated using Mega v.7.0 [84] under the general settings of the selected models with 1000 bootstraps.

Identification of barriers to gene-flow between populations of *U. crassus* was conducted using Barrier v2.2 [65]. The geographical map was created with a dual structure of the Voronoi diagram [85], the Delaunay triangulation method [86], which allowed populations with a set of triangles to be connected. This analysis was based on coordinates of the sampling sites. The Fst values calculated by Arlequin v.3.5. were used as a matrix data to link computational geometry of the Delaunay network. Finally, in the geometric network, genetic boundaries (in hierarchical order, from “a” to “e”) were identified and calculated according to the Monmonier’s maximum difference algorithm [87].

For estimating ancestral population dynamics through time on the basis of mtDNA sequences, BEAST 1.7. was used [88]. BEAST uses standard Markov chain Monte Carlo (MCMC) sampling procedures to estimate a posterior distribution of effective population size through time directly from a sample of gene sequences, given any specified nucleotide substitution model. The data set for this analysis consisted of the alignment of all obtained sequences, not just unique haplotypes. Three demographic models: constant size, exponential population growth, and Bayesian Skyline were tested. Each of these was run with the eight possible site models: HKY or GTR (General Time Reversible) with either equal rates, proportion of invariable sites or gamma distributed variable rates. To ensure convergence and achieve a high Effective Sample Size (ESS) value (at least 300) for every estimated parameter, each analysis was run at least in quadruplicate. After examination of the log files in Tracer [88], the results from all the runs were combined in LogCombiner 1.7.5 [88], for each model respectively, removing the non-stationary burning data. The best demographic and site models were a posteriori selected, through Bayes Factor comparisons, using the method of Newton and Raftery (1994) with the modification proposed by Suchard, Weiss, and Sinsheimer (2001) as implemented in Tracer [89,90]. The models implementing exponential population growth were slightly better than constant size or the bulk synchronous parallel (BSP) models, but without significant differences.

## 3. Results

Bearing in mind that all three genes could not be analyzed in all specimens, 243 sequences (83 *cox1*, 95 *ndh1,* and 65 ITS region) were obtained for the 99 specimens examined (Table S1). Only somatic tissue was sampled, and there was no evidence of heteroplasmy or doubly uniparental inheritance DUI [91].

The partition homogeneity test (as implemented in PAUP) showed no significant differences between the phylogenies reconstructed from *cox1* and *ndh1* (P = 0.35), such that the data sets could be combined.

### 3.1. Mitochondrial Gene Regions

The alignment of the two combined mitochondrial regions of *U. crassus* produced 1473 characters (614 for *cox1* and 859 for *ndh1*), 46 of which were variable and 41 parsimony informative. 

The results of mtDNA polymorphism analyses and relative frequencies of haplotypes for separate and combined mtDNA data are presented in Table 1. Seventeen mitochondrial haplotypes (h = 0.688, π = 0.012) were found among 79 individuals of *U. crassus* from 12 localities in Poland and Lithuania where sequences for both genes (*cox1* and *ndh1*) were available. 

Below, we present the results of analyses for combined mtDNA data and selected results for both mitochondrial regions (*cox1* and *ndh1*) analyzed separately. 

### 3.2. Within Population Variability

Analyses of within population structure revealed a high number of mtDNA haplotypes unique to a particular population (Table 1). In the case of mtDNA haplotype richness, Polish and Lithuanian populations averaged 2.7 and 2.3 haplotypes respectively (Table 1). The sequence divergence within- population was rather low with a maximum value (π = 0.014) for Pilica (Table 1).

### 3.3. Among Population Structure

For mtDNA data, only one haplotype (CN6) was observed in populations from both drainages—Vistula and Neman. Only one haplotype (CN1) was shared among all Polish populations but we did not find any haplotype common to all Lithuanian populations. However, in the case of *ndh1* polymorphism, N4 and N8 haplotypes were observed in almost all Lithuanian populations (with the exception of Virvicia). The relative frequencies of shared mtDNA haplotypes varied from 0.083 to 1.000 (Table 1).

A hierarchical AMOVA conducted over the 12 populations of the two drainages indicated that approximately 77% of the variation arose from genetic variation between populations from the Neman and Vistula drainages (Φ_DT_ = 0,771; P = 0.003), whereas only about 4% of the variation was due to differences among populations within drainages (Φ_SD_ = 0.189; P < 0.001). Correspondingly, there was significant mtDNA structure among all *U. crassus* populations (Φ_ST_ = 0.813; P < 0.001). 

Comparison of population differentiation within drainages for mtDNA showed also some genetic structure among populations within the Vistula drainage (Φ_ST_ = 0.221; P = 0.007). However, the estimated level of diversity was much lower than in the case of between drainages comparison.

The population structure occurred also on the finest spatial scale: between populations from river Pilica and its tributary Czarna Włoszczowska (Φ_ST_ = 0.181 but P = 0.100). Interestingly, strong genetic differentiation also occurred between populations from rivers located in Central and Southern Poland belonging to the Middle and Upper Vistula drainages, respectively (Φ_ST_ = 0.302, P = 0.000).

In the case of the Neman drainage, the Φ_ST_ estimate value (Φ_ST_ = 0.146) indicated a moderate level of genetic structure among analyzed populations, however the value was not significant (P = 0.107).

**Figure 2:**
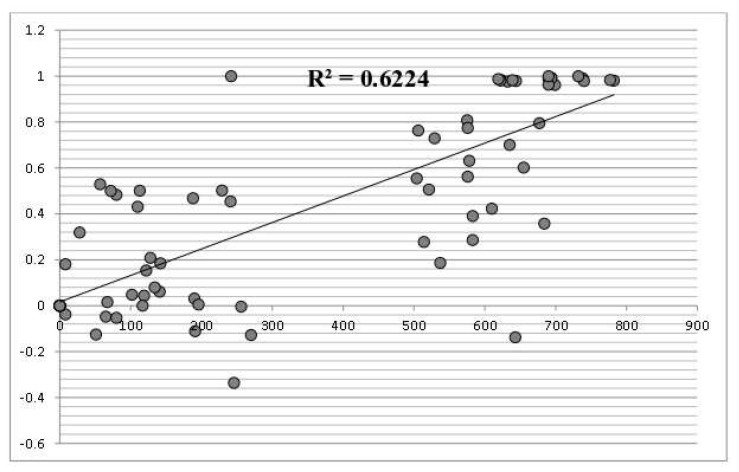
Results of Mantel tests between pairwise geographic distance among sampling sites in km (x axis), and pairwise Fst estimates (y axis) using mtDNA haplotypes.

Pairwise Φ_ST_ sample comparisons and gene flow estimations (Nm) between populations are summarized in Table 2. The Mantel’s test results (Figure 2) indicated, in general, a significant relationship between pairwise Φ_ST_ estimates and geographical distances for comparisons among all locations (r^2^ = 0.622, P < 0.001). However, in some cases it was observed that pairwise Φ_ST_ estimates did not increase with geographic distance between sampling sites, and distant populations did not show higher genetic structure (i.e., populations from Pilica and Luknelis representing different drainages; Table 2). On the contrary, as already mentioned adjacent populations from Pilica and its tributary Czarna Włoszczowska showed significant differentiation (Φ_ST_ = 0.181; Table 2).

**Figure 3:**
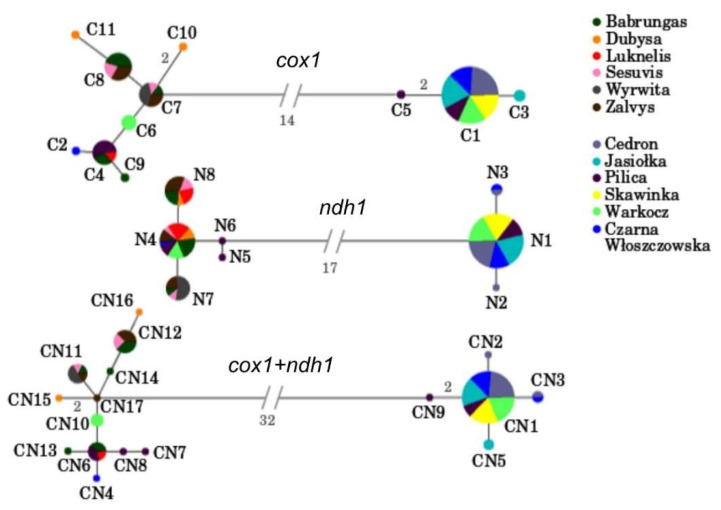
Median-joining network based on mtDNA sequences of *U. crassus*. The size of the circles is proportional to the number of sequences. The mutational steps values are indicated along the lines.

Mismatch analysis of mtDNA haplotypes showed an average of 8.16 bp difference between sequences (1.81% sequence difference), within a range of 1–32 bp difference. These haplotype differences are mirrored in network reconstructions (Figure 3) where we found two evolutionary distinct groups of haplotypes separated by a high number of mutational steps (32 for combined mtDNA data). On the contrary, haplotypes within each of the groups were separated by only one or two mutational steps. Most individuals from Vistula drainage represented one haplotype. The observed distribution of mtDNA haplotypes among *U. crassus* populations from different localities in most cases is congruent with geographical subdivision into Vistula and Neman drainage. The only exceptions are mtDNA haplotypes from several individuals found in central Poland rivers (PIL, CZW, WAR) that clustered with haplotypes from Lithuanian populations.

**Figure 4:**
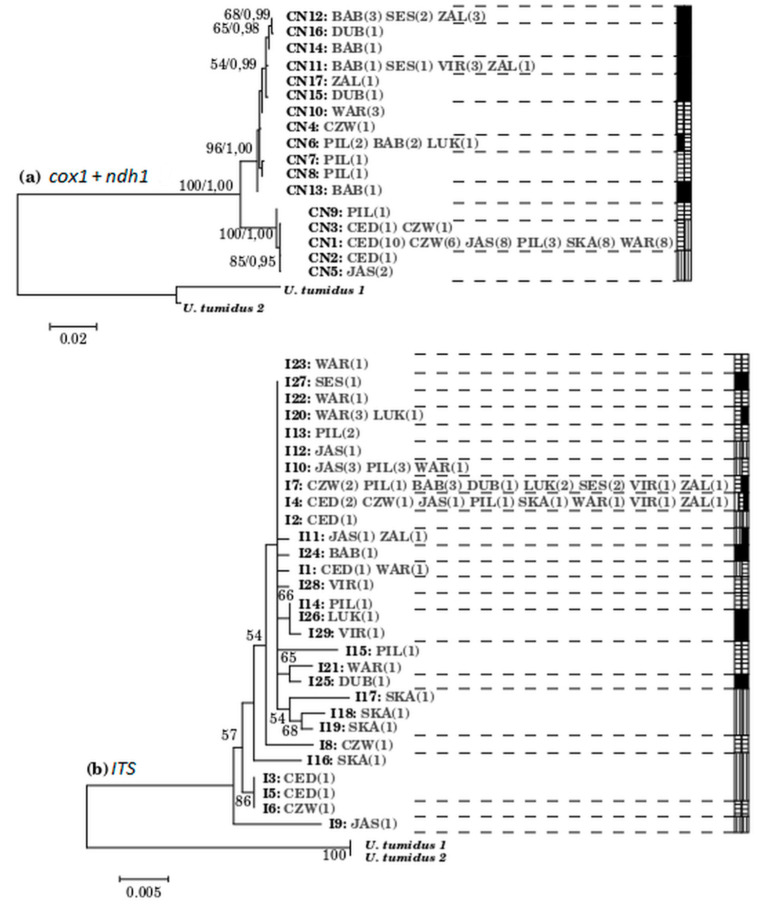
Phylogenetic ML trees of *U. crassus* haplotypes. (**a**) based on concatenated mitochondrial data (*cox1* + *ndh1* regions); (**b**) based on the entire ITS region of the nuclear ribosomal DNA variants of *U. crassus*; gaps were included. The trees were rooted with *Unio tumidus* (Acc. Nos: KJ525923–KJ525927; KJ525965, KJ525966). Bootstrap values higher than 50 are given next to the respective node. The scale bar indicates the number of substitutions per site. Locality codes: CED–Cedron, CZW–Czarna Włoszczowska, JAS–Jasiołka, PIL–Pilica, SKA–Skawinka, WAR–Warkocz, BAB–Babrungas, DUB–Dubysa, LUK–Luknelis, SES–Sesuvis, VIR–Virvita, ZAL–Zalvys. Unique haplotypes were given Arabic numbers. Numbers in brackets indicate the number of individuals from a particular locality. Colors depict geographical regions: Lithuania (black), Central Poland (grey), and Southern Poland (white).

The topology of the ML phylogenetic tree based on concatenated mtDNA data revealed evident phylogeographic structure with strong bootstrap support (Figure 4a). First clade (clade 1) consisted only of haplotypes from all Polish localities (Vistula drainage) whereas in the second (clade 2) haplotypes from both drainages were intermingled. However only haplotypes from the central Poland region: Pilica, its tributary—Czarna Włoszczowska and Warkocz (PIL, CZW, WAR)—were found in the second clade together with Lithuanian haplotypes. The southern Poland haplotypes from Cedron, Jasiołka, and Skawinka (CED, JAS, SKA) were grouped in the first clade. So, neither Polish nor Lithuanian populations relating to the Vistula and Neman rivers, respectively formed monophyletic clade.

Genetic discontinuities between populations of *U. crassus* were identified as barriers to gene-flow (Figure 1). Using Monmonier’s maximum difference algorithm we indicated two significant barriers, described as “a” and “b”. Furthermore, three other barriers were included, indicated as “c”, “d”, and “e”, showing where gene-flow was also limited (Figure 1). The first main predicted barrier (“a”) separated all populations from the Vistula drainage (Poland) and the Neman drainage (Lithuania). The second main barrier (“b”) separated populations from Vistula drainage according to their geographical localization: Middle and Upper Vistula drainages.

**Figure 5:**
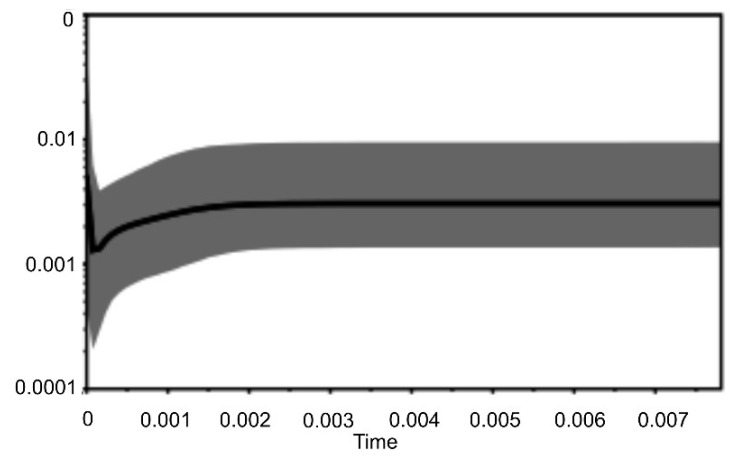
Bayesian skyline plot derived from a set of mtDNA sequences of *U. crassus*. The x axis is in units of time (mutation per site), and the y axis is equal to Neτ (the product of the effective population size and the generation time in mutational units). The median estimates are shown as thick black line, the 95% highest posterior density (HPD) limits are shown by the grey areas.

The BSP method revealed a flat plot without fluctuation with a relatively recent decrease and rapid increase in the effective population size of *U. crassus* (Figure 5). The apparent decline started at the time needed to accumulate 0.0015 substitutions per site in the analyzed combined mtDNA fragments. The rapid rise started near to 0.0001 substitutions per site. The calculation of the approximate dates, when these two demographic events took place was done according to the molecular rate for the order Unionida (0.265 ± 0.06% per million years) estimated by Froufe et al. (2016) [92]. According to these estimates the decline could have started in the Middle Pleistocene (ca. 566 ka BP), while the rapid incline in the Late Pleistocene, during the Weichselian glaciation (ca. 38 ka BP), before the Last Glacial Maximum.

### 3.4. The Entire ITS Region of the Nuclear Ribosomal DNA

The alignment of the nrDNA region produced 879 characters and possessed one 11-bp, one 6-bp, and several 1- to 2-bp separate indels. The number of variable and parsimony informative characters was 42 and 13 respectively when gaps were treated as missing information. 

The results of nrDNA polymorphism analyses (gaps considered) are presented in Table 1. In total, 29 nuclear sequence variants (h = 0.919, π = 0.006) were found among 62 individuals of *U. crassus* from 12 localities in Poland and Lithuania (Table 1). When sites with gaps were excluded, the number of nuclear sequence variants was reduced to 19 (h = 0.602, π = 0.054, data not shown). 

Similar to mtDNA data, we observed nrDNA variants shared by Lithuanian populations and populations from Central Poland (PIL, CZW, WAR): I7 and I20 (Table 1). Interestingly, there were also cases of nrDNA variants found in Lithuanian as well as southern Polish populations: I4 and I11 (Table 1). I4 was shared among all Polish populations and two Lithuanian ones and the other way round—I7 was shared among all Lithuanian and two Polish ones. There was one more nrDNA variant not unique for a single population (Table 1): I10–shared among JAS, PIL, and WAR. The relative frequencies of shared variants varied from 0.111 to 0.750 (Table 1) in particular populations. The haplotype diversity (h) within populations (Table 1) ranges from 0.500 (BAB) to 1.000 (SKA, VIR).

The same topology of the ML phylogenetic tree (Figure 4b) was found regardless of how gaps were treated, but the support values slightly increased when the information from gaps was included. The phylogenetic tree obtained by the ML method did not reveal any evident phylogeographic structure and bootstrap supports for branches were rather low (Figure 4b). The nrDNA sequences variants from different localities within Vistula and Neman drainages were intermingled. Only I9 which possessed 11-bp insertion, occupied an isolated position in the tree. 

## 4. Discussion

The relationship between dispersal and differentiation of European freshwater mussels in the drainage scale has been barely studied so far. Nagel (2000) [93] investigated genetic relationships within *Unio pictorum* (Linnaeus, 1758) from central Europe on the basis of geographical distribution of allele frequencies at 17 enzyme loci. His results in general show that the genetic affinities of the populations are the closest within the same drainage basin. Similarly, North American unionid species show the same pattern of relationships between genetic diversity and the history of drainage system ([93] and references therein). In the present study, a hierarchical AMOVA also indicated that only 4% of variation was due to differences among *U. crassus* populations within drainages while genetic differences between the Neman and Vistula drainages were strongly indicated (77% of variation). Genetic variation was different in the results reported by Feind et al. (2018) [53]. They characterized the genetic constitution of 18 *U. crassus* populations in Germany and Sweden originating from six major drainage systems (Elbe, Rhine, Danube, Schlei-Trave, Eider and Kävlingeåns). Only 9.1% of the variation was due to differences among drainage systems; 6.9% of the variation was explained by differences among populations within drainage systems. Analyses performed on populations of other endangered freshwater mussel, *M. margaritifera*, show different values of genetic differentiation within drainages [5], the lowest in the case of the Elbe and Danube drainages (Fst = 0.121 and Fst = 0.240, respectively) and the highest for the Meuse river (Fst = 0.773). For comparison, in the Vistula and Neman drainages we obtained Φ_ST_ = 0.221 and Φ_ST_ = 0.146, respectively. However, the global fixation index for all the Polish and Lithuanian populations of *U. crassus* studied here was more than twice as high as that obtained by Geist and Kuehn (2005) [5] for *M. margaritifera* (Φ_ST_ = 0.813 versus Fst = 0.374, respectively). It suggests that in the case of *U. crassus* we dealt with at least two different genetic units.

The boundary between genetic diversity patterns of *U. crassus* populations from the Vistula drainage (Poland) and those from the Neman drainage (Lithuania) was revealed in our study by the level of mtDNA markers. Although some minor inconsistencies were observed between genetic diversity patterns revealed by mitochondrial and nuclear markers, the occurrence of two well-defined groups associated with the two considered drainages was fairly well supported (Figure 3 and Figure 4). Independent sources of characters, such as mitochondrial and nuclear DNA, can reflect different, but equally accurate, phylogenetic patterns (e.g., [94,95,96,97,98,99]). Nevertheless, such incongruence between these two data sources does not provide a criterion by which to select one phylogeny over another. In fact, these disagreements often lead to an enhanced understanding of the evolutionary history of the species in question, including elucidation of patterns of introgression, complex population structure or sex- biased gene flow [100]. Therefore, we suggest that minor differences in relationships among mitochondrial and nuclear haplotypes described here are not the limitation for the proposed inference about relationships of the tested *U. crassus* populations. However, despite already postulated utility of ITS sequences to investigate phylogenetic relationship within Unionidae [12], we did not fully support this idea—the ITS analyses should be backed by investigation of the other genetic markers. 

In the study described here, with the few exceptions we identified two distinct groups of *U. crassus* populations inhabiting rivers belonging to two different drainage basins. The results suggest that different evolutionary paths of the analyzed populations may be one of the components that differentiate the genetic units identified here. Another one could be the geographic isolation of populations from various river basins that lead to reduced gene flow and, at the same time, greater influence of genetic drift on genetic structure. The reduction of gene flow promoting genetic differentiation of populations may also be caused by habitat fragmentation due to anthropogenic factors [91]. Here, such an explanation may be proposed in the case of *U. crassus* populations from rivers representing central Poland: Pilica (flowing into the middle Vistula) and its tributary, the Czarna Włoszczowska river. High Φ_ST_ values suggest reduction of gene flow between particular populations, although we should expect them to share the same haplotypes. On the contrary, these populations share only one mtDNA haplotype that is also present in the rest of the Polish populations whereas the remaining haplotypes are unique for each population (Table 1). Studies on other mussel species also indicate the isolation of populations in neighboring river basins and even the lack of gene flow between populations from different tributaries of the same river (e.g., [27]). Moreover, Geist and Kuehn (2005) [5] found a high fragmentation level of the structure of freshwater pearl mussel (*M. margaritifera*) populations inhabiting five main European river basins, the effect of the limited gene flow between these populations. Zanatta, Fraley, and Murphy (2007) [101] in turn observed a high gene flow between populations of a unionid wavy-rayed lampmussel (*Lampsilis fasciola* Rafinesque, 1820) inhabiting the same river basin, indicating the presence of panmixia in this species. Elderkin et al. (2007) [32] also used a hierarchical approach to study population genetic structure of other unionid mussel *Amblema plicata* (Say, 1817) among rivers and drainages. Interestingly, they found low genetic structure among rivers and drainages separated by large geographic distances, what may indicate high effective population size and/or highly agile fish hosts for this species. In the case of *U. crassus* populations in this study, the Mantel test revealed a relatively strong relationship between Φ_ST_ estimates and geographical distances (Figure 2). However, in detail, the pattern of genetic diversity seems to be much more complicated. Therefore, we suggest that the observed pattern of *U. crassus* genetic diversity distribution has been shaped by different factors, i.e. historical and current gene flow.

The distribution of the genetic diversity should reflect the present or past connections within and between drainage systems. Results obtained for *U. crassus* populations in this work partly fulfill this hypothesis as most of the sample populations cluster according to their distribution within the two analyzed drainages. However, there are some mtDNA haplotypes found in central Poland populations (PIL, CZW, WAR) that clustered with haplotypes from Lithuanian populations. Nagel (2000) [93] also found exceptions from general congruence between spatial and genetic distances in the case of European populations of a painter’s mussel *U. pictorum*. Similar results were obtained also by Geist and Kuehn (2005) [5] for populations of *M. margaritifera* separated only by 20 km in geographical distance within the same drainage, which were located in different clades on the cladogram. Also, populations from the Danube did not cluster together. Two possibilities were proposed by the authors to explain these irregularities—first, the canals connecting rives and alternatively, tectonic movements affecting the river systems. In our case, the role of canals in promoting gene flow between populations can be seen in the case of the *U. crassus* population from the Babrungas river (tributary of Venta) connected with the Dubysa river by the Windawski Canal of only 15 km in length. Both, Babrungas and Dubysa populations share the same mtDNA haplotypes. On the contrary, the Augustowski Canal connecting Vistula and Neman drainages through the Biebrza river—A tributary of the Narew river, and the Neman river through its tributary, the Czarna Hańcza river—does not seem to influence the observed pattern of genetic diversity of *U. crassus*. However, further investigations including populations of *U. crassus* from northeastern Poland should be conducted to provide full support for this claim. 

Humans have profoundly influenced most aquatic ecosystems. For example, formerly separate drainage systems were connected by canals promoting faunal exchange across long established boundaries. At present, anthropogenic factors strongly influence fauna, causing species extinction, changing current distribution ranges, enhancing invasions of alien species that force out indigenous species, or reducing genetic diversity. The anthropogenic pressure, which has caused fragmentation of *U. crassus* habitat and population declines, might well shape consequently genetic architecture and distribution of mtDNA haplotypes of this mussel. As stated by Zając (2004) [43], populations of this mollusk are in Poland quite often isolated and scattered, as well as the overall degree of *U. crassus* preservation is improper mainly due to small population size and unfavorable status of the mussel habitats. Small populations, in turn, are more susceptible to the effect of inbreeding and genetic drift. In our study we included populations reported as declined due to human activity and/or isolated by polluted fragments of rivers at different time scales. Such a population is Jasiołka (JAS), characterized by the presence of one common mtDNA haplotype (CN1) found also in the remaining Polish populations and one private mtDNA haplotype (CN5) (vide Table 1). Besides, human activities such as commercial trade with live fish are believed to influence their genetic composition [93]. In turn, fragmentation of the environment is a barrier to the movement of organisms and is therefore a major threat to species existence both for demographic and genetic reasons [102]. Thus, it can be inferred that the pattern of genetic variability of freshwater mussels and of *U. crassus* is constantly changing. In turn, if current water connections and ongoing gene flow are the main forces driving patterns of genetic diversity of *U. crassus*, populations located nearby (e.g., Skawinka with its tributary Cedron; Pilica with its tributary Czarna Włoszczowska) should be similar genetically and share the same haplotypes of mtDNA whereas the Lithuanian populations should be different from the Polish ones. However, we found some mtDNA haplotypes found in central Poland populations (PIL, CZW, WAR) that clustered with haplotypes from Lithuanian populations (Figure 4). Therefore, we suggest that historical phenomena could most strongly shape genetic diversity of *U. crassus* in Europe.

Pleistocene ice ages have exerted considerable influence on the contemporary genetic diversity patterns of freshwater species. Multiple transgressions and regressions of ice caused the situation that many species had to displace or escape to the ice age refugia. In glacial periods, the northern part of Poland and the area of Lithuania were covered to varying degrees by the glacier (for example, during the Sanian 2 glaciation, this area was almost entirely covered with ice sheets that reached the Carpathians), causing the withdrawal of organisms from this region [103]. Glacial water outflows in different directions also occurred in the Pleistocene, e.g., from the area of the current San and Pilica river basins to the east, to the Dniester river valley [103]. The merging of these river systems has only subsided as a result of the final formation of the Vistula River. In addition, water from the Neman basin drained southwest to the Torun-Eberswalder Urstromtal during the Pomeranian phase of the Vistulian Glaciation. Then, after the glacier retreat, rivers of northern Poland and Lithuania started to flow towards the Baltic Sea (e.g., [104]). In the case of Polish and Lithuanian populations of *U. crassus* the observed intermingling of haplotypes may be connected to the ancient connection between Neman and Vistula drainages as indicated by the geological data. During the Vistulian Glaciation the ice sheet blocked the pre-existing drainage system and caused the development of vast ice-dammed lakes including waters of the ancient Neman River as well as the Polish rivers Biebrza, Narew, and Vistula [103]. Pre-existing connection between the Neman and Vistula waters seems to be also confirmed by morphological similarities between ichtiofauna of their drainages [105]. In turn, if current water connections and ongoing gene flow are the main forces driving patterns of genetic diversity of *Unio* populations, populations located nearby (e.g., Skawinka with its tributary Cedron; Pilica with its tributary Czarna Włoszczowska) should be similar genetically and share haplotypes of mtDNA whereas Lithuanian populations should be different from Polish ones. The genetic diversity and differentiation of *U. crassus* populations revealed during this study can also be explained by colonization from different glacial refugia or postglacial recolonization. Moreover, the observed pattern of genetic diversity may result from specificity between glochidia and host fish vectors.

As larval stages of unionids (glochidia) are obligate parasites of freshwater fish, they were able to migrate and recolonize the European areas during warmer periods only along inland waters together with their host fish species. Thus, the gene flow among populations of freshwater mussels was mostly affected by migration patterns of fish host species. Potential hosts for *U. crassus* include fish species like bullhead (*Cottus gobio* Linnaeus, 1758), Eurasian Minnow (*Phoxinus phoxinus* Linnaeus, 1758), chub (*Squalius cephalus* Linnaeus, 1758), rudd (*Scardinius erythrophthalmus* Linnaeus, 1758) Three-spinned Stickleback (*Gasterosteus aculeatus* Linnaeus, 1758), and perch (*Perca fluviatilis* Linnaeus, 1758) (e.g., [106,107,108]).

The Ponto-Caspian region (the Black Sea, Sea of Azov and the Caspian Sea) was the main glacial refugium of the recent fish fauna of both Poland and Lithuania [109] which colonized northern Europe through two main river networks: Dniester-San-Vistula and Dnieper-Neman-Vistula [110]. The pattern of drainage systems in Central Europe changed many times in the Pleistocene, but the major route of recolonization of this part of Europe became the Dniester and Dnieper (northern trail) because several species were not able to use the Danube (southern trail) due to the barrier of the Carpathians Mountain Range. The colonization of Europe by fish-hosts of *U. crassus* has been the subject of many studies, for example: *Squalius cephalus* [111], *Perca fluviatilis* [112], *Cottus gobio* [113,114], *Gasterosteus aculeatus* [115]. Nevertheless, it is difficult to point out the simple relationship between the hypothetical postglacial migration pathways of any fish species and the distribution of *U. crassus* evolutionary lines. However, the *U. crassus* expansion pattern has some characteristics of the observed distribution of other species of freshwater mussels. For example, the analysis of DNA microsatellite loci of *M. margaritifera* from central Europe revealed that the diversity of individual populations is not consistent with the modern hydrological network [5]. The genetic variability pattern of *M. margaritifera* was also not compatible with the distribution of genetic variability of *C. gobio*, one of the fish-host species of glochidia in the same area [116]. These differences, however, are not surprising given the possibility of dispersal of the freshwater pearl mussel through other fish hosts, whose migration paths are different (e.g., [117]). A study of Berg et al. (1998) [118] indicates that a varied level of population migration can be expected even within the same river basin. The observed complex pattern of genetic variability distribution was thus explained by the influence of glacial phenomena and links between the presently separated river basins existing in the past [119]. Interestingly, Nagel (2000) [93] presented the dendrogram, based on Nei’s genetic distance, in which individuals of *U. pictorum* from the Vistula are separated from those inhabiting other rivers of the Baltic Sea catchment and cluster with populations of the Danube. Similarly, geographic isolation was found in the present study between populations belonging to the same river basin, which also indicated the existence of hydrological barriers in migrations of glochidia hosts. Therefore, we suggest that the genetic relationships within *U. crassus* from Poland and Lithuania reflect paleogeographical relationships between river systems during Pliocene and Pleistocene rather that current gene flow.

Interestingly, in the case of our study the present-day population differentiation of *U. crassus* did not match the present-day drainage systems in the case of the central Poland rivers. Individuals from the southern Poland rivers were localized in “Polish cluster” and did not intermingle with Lithuanian populations. The observed pattern of genetic diversity and differentiation of *U. crassus* populations revealed during this study can be explained either by historical gene flow or different routes of post glacial colonization. In such a case, the region of central Poland could be a contact zone between two haplotype lineages. 

We believe that our results suggest the existence of a secondary contact area in central Poland resulting from the recolonization of a given area by populations from separate glacial refugia [120]. The identified suture zone is at the same time a barrier to further expansion of genealogy lines from different glacial refugia and maintains their integrity. Different populations from different evolutionary lines can be distinguished in populations present in the suture zone due to the presence of various haplotypes [121]. In Europe, there are five identified suture zones: eastern and western Europe, central Europe, central Scandinavia, the Alps, and the Pyrenees [120,122,123]. Poland is also a specific suture zone, characterized by the presence of multiple secondary contact zones, distinguished for different refugial lines (compare e.g., data on weasel *Mustela nivalis* Linnaeus, 1758 by McDevitt et al., 2012 [124]). 

It is worth mentioning that for *U. crassus,* a number of subspecies with local forms of uncertain taxonomic rank has been widely accepted [125,126,127,128]. So, such a high level of genetic diversity between specimens from the Vistula and Neman drainages may support the above-mentioned idea. On the other hand, the results of this study revealed mtDNA haplotype shared by specimens form both drainages and some Polish haplotypes cluster together with the Lithuanian ones. Therefore, the observed genetic structure does not match the present drainage systems, so there is no straight correlation between genetic diversity and geographic region.

In conclusion, the results of our study indicated that in the case of eastern Central European populations of *U. crassus* we dealt with at least two different genetic units. However, the observed genetic structure does not match the present drainage systems. Therefore, we suggest that the observed genetic relationships within *U. crassus* from Poland and Lithuania reflect rather paleogeographical relationships between river systems during the Pliocene and Pleistocene than being the result of the current gene flow. The present-day genetic pattern of *U. crassus* diversity may also be shaped by different routes of post glacial colonization and specificity between glochidia and host fish vectors. More recent habitat fragmentation due to anthropogenic factors may also have contributed to the observed populations structure. 

## Figures and Tables

**Table 1:** Polymorphism estimates and relative frequencies of haplotypes for separate and combined mtDNA data of *U. crassus* from the studied localities. Locality code: CED–Cedron, CZW–Czarna Włoszczowska, JAS–Jasiołka, PIL–Pilica, SKA–Skawinka, WAR–Warkocz, BAB–Babrungas, DUB–Dubysa, LUK–Luknelis, SES–Sesuvis, VIR–Virvita, ZAL–Zalvys; N–number of individuals; n–number of haplotypes; π–nucleotide diversity; HD (SE)–standard error.

Haplotype	Vistula Drainage						Neman Drainage					
*cox1*	PIL	CZW	WAR	CED	SKA	JAS	BAB	DUB	LUK	SES	VIR	ZAL
**C1**	0.500	0.875	0.727	1.000	1.000	0.833						
**C2**		0.125										
**C3**						0.167						
**C4**	0.400						0.250		1.000			
**C5**	0.100											
**C6**			0.273									
**C7**							0.125			0.333	1.000	0.400
**C8**							0.500			0.667		0.600
**C9**							0.125					
**C10**								0.500				
**C11**								0.500				
**N**	10	8	11	12	8	12	8	2	1	3	3	5
***n***	3	2	2	1	1	2	4	2	1	2	1	2
**HD**	0.644	0.250	0.436	0.000	0.000	0.303	0.750	-	-	0.667	0.000	0.600
**(SE)**	(0.101)	(0.180)	(0.133)			(0.147)	(0.139)			(0.314)		(0.175)
**π**	0.016	0.008	0.012	0.000	0.000	0.001	0.003	-	-	0.001	0.000	0.001
**(SE)**	(0.009)	(0.005)	(0.007)			(0.001)	(0.002)			(0.001)		(0.001)
***ndh1***	**PIL**	**CZW**	**WAR**	**CED**	**SKA**	**JAS**	**BAB**	**DUB**	**LUK**	**SES**	**VIR**	**ZAL**
**N1**	0.500	0.750	0.727	0.833	1.000	1.000				0.200		
**N2**				0.083								
**N3**		0.125		0.083								
**N4**	0.200	0.125	0.272				0.500	0.667	0.571	0.200		0.250
**N5**	0.100											
**N6**	0.100											
**N7**							0.125			0.200	1.000	0.250
**N8**							0.375	0.333	0.429	0.400		0.500
**N**	9	8	11	12	9	10	8	3	7	5	5	8
***n***	4	3	2	3	1	1	3	2	2	4	1	3
**HD**	0.694	0.4643	0.436	0.318	0.000	0.0000	0.679	0.667	0.571	0.900	0.000	0.714
**(SE)**	(0.147)	(0.200)	(0.133)	(0.164)			(0.122)	(0.314)	(0.119)	(0.161)		(0.123)
**π**	0.012	0.006	0.009	0.001	0.000	0.000	0.001	0.001	0.001	0.010	0.000	0.001
**(SE)**	(0.007)	(0.003)	(0.005)	(0.001)			(0.001)	(0.001)	(0.001)	(0.006)		(0.001)
***cox1*+ *ndh1***	**PIL**	**CZW**	**WAR**	**CED**	**SKA**	**JAS**	**BAB**	**DUB**	**LUK**	**SES**	**VIR**	**ZAL**
**CN1**	0.375	0.750	0.727	0.833	1.000	0.800						
**CN2**				0.083								
**CN3**		0.125		0.083								
**CN4**		0.125										
**CN5**						0.200						
**CN6**	0.250						0.250		1.000			
**CN7**	0.125											
**CN8**	0.125											
**CN9**	0.125											
**CN10**			0.273									
**CN11**							0.125			0.333	1.000	0.200
**CN12**							0.375			0.667		0.600
**CN13**							0.125					
**CN14**							0.125					
**CN15**								0.500				
**CN16**								0.500				
**CN17**												0.200
**N**	8	8	11	12	8	10	8	2	1	3	3	5
***n***	5	3	2	3	1	2	5	2	1	2	1	2
**HD**	0.857	0.464	0.436	0.318	0.000	0.356	0.857	-	-	0.667	0.000	0.700
**(SE)**	(0.108)	(0.200)	(0.133)	(0.164)		(0.159)	(0.108)			(0.314)		(0.218)
**π**	0.014	0.007	0.011	0.000	0.000	0.000	0.002	-	-	0.001	0.000	0.001
**(SE)**	(0.008)	(0.004)	(0.006)	(0.000)		(0.000)	(0.001)			(0.001)		(0.001)

**Table 2:** The estimates of Φ_ST_ (below diagonal) and number of migrants (Nm, above diagonal) between pairs of populations of *U. crassus* from different localities. Locality code: CED–Cedron, CZW–Czarna Włoszczowska, JAS–Jasiołka, PIL–Pilica, SKA–Skawinka, WAR–Warkocz, BAB–Babrungas, DUB–Dubysa, LUK–Luknelis, SES–Sesuvis, VIR–VIRVITA, ZAL–Zalvys. Fairy gray background indicates Vistula drainage; estimates between Skawinka with its tributary Cedron and Pilica with its tributary Czarna Włoszczowska were marked in bold. Dark grey background indicates Neman drainage. * *p* < 0.05.

Locality Code	CED	CZW	JAS	PIL	SKA	WAR	BAB	DUB	LUK	SES	VIR	ZAL
**CED**		10.726	7.635	10.726	**inf**	1.889	0.018	0.010	0.005	0.008	0.003	0.009
**CZW**	0.044		15.139	**2.263**	inf	inf	0.118	0.184	0.213	0.155	0.146	0.128
**JAS**	0.061	0.032		0.567	5.714	2.203	0.019	0.012	0.005	0.009	0.004	0.010
**PIL**	0.502 *	**0.181**	0.469 *		0.658	31.104	0.776	2.185	inf	1.304	1.252	0.898
**SKA**	−0.038	0.000	0.080	0.432 *		2.750	0.019	0.008	0.000	0.006	0.000	0.008
**WAR**	0.209 *	−0.047	0.185	0.016	0.154		0.291	0.486	0.682	0.400	0.389	0.330
**BAB**	0.966 *	0.809 *	0.962 *	0.392 *	0.963 *	0.632 *		9.900	inf	inf	1.066	inf
**DUB**	0.980 *	0.730	0.977 *	0.186	0.982 *	0.507 *	0.048		inf	inf	0.536	114.931
**LUK**	0.991	0.701	0.990	−0.137	1.000	0.423	−0.128	−0.112		0.603	0.000	0.445
**SES**	0.984 *	0.764 *	0.982 *	0.277 *	0.988 *	0.555 *	−0.053	−0.126	0.453		0.500	inf
**VIR**	0.992 *	0.774 *	0.992 *	0.285 *	1.000 *	0.562 *	0.319	0.482	1.000	0.500		0.495
**ZAL**	0.981 *	0.796 *	0.979 *	0.358 *	0.984 *	0.602 *	−0.004	0.004	0.529	−0.336	0.502	

## Supplementary Materials

The following are available online at https://www.mdpi.com/2075-1729/10/7/119/s1, Table S1: Information on specimens of *Unio crassus* under study and Table S2. Data on sampling localities from Poland and Lithuania.
