# Supplementary material for: The Patterns and Puzzles of Genetic Diversity of Endangered Freshwater Mussel Unio crassus Philipsson, 1788 Populations from Vistula and Neman Drainages (Eastern Central Europe)
Source: Life (Basel). 2020 Jul 21;10(7):119. doi: 10.3390/life10070119 (PMC7400583; doi:10.3390/life10070119)
Supplement: Supplementary file 1 [file life-10-00119-s001.pdf]

Table S1. Information on specimens of *Unio crassus* under study

**River (locality code) / Drainage**	**Country**	**Individuals**	**Haplotypes of *cox1*** (GenBank accession number)	**Haplotypes of *ndh1*** (GenBank accession number)	**Haplotypes of *cox1+ ndh1***	**Sequence variation at *ITS* region** (GenBank accession number)
**PILICA (PIL) / MIDDLE VISTULA RIVER**	**POLAND**	6P	C1 (KJ525912.1)	-	-	-
		7P	C1 (KJ525912.1)	-	-	-
		10P	C1 (KJ525912.1)	N1 (KJ525928.1)	CN1	I7 (KJ525942.1)
		11P	C4 (KJ525915.1)	N4 (KJ525931.1)	CN6	I4 (KJ525939.1)
		12P	C1 (KJ525912.1)	N1 (KJ525928.1)	CN1	I13 (KJ525948.1)
		13P	C4 (KJ525915.1)	N5 (KJ525932.1)	CN7	I14 (KJ525949.1)
		16P	C4 (KJ525915.1)	N6 (KJ525933.1)	CN8	I13 (KJ525948.1)
		19P	C5 (KJ525916.1)	N1 (KJ525928.1)	CN9	I10 (KJ525945.1)
		21P	C4 (KJ525915.1)	N4 (KJ525931.1)	CN6	I15 (KJ525950.1)
		22P	C1 (KJ525912.1)	N1 (KJ525928.1)	CN1	I10 (KJ525945.1)
		23P	-	N1 (KJ525928.1)	-	I10 (KJ525945.1)
**CZARNA WŁOSZCZOWSKA (CZW) / MIDDLE VISTULA RIVER**	**POLAND**	3CW	C1 (KJ525912.1)	N1 (KJ525928.1)	CN1	I4 (KJ525939.1)
		5CW	C1 (KJ525912.1)	N1 (KJ525928.1)	CN1	I6 (KJ525941.1)
		6CW	C1 (KJ525912.1)	N3 (KJ525930.1)	CN3	-
		14CW	C1 (KJ525912.1)	N1 (KJ525928.1)	CN1	I7 (KJ525942.1)
		15CW	C2 (KJ525913.1)	N4 (KJ525931.1)	CN4	I8 (KJ525943.1)
		16CW	C1 (KJ525912.1)	N1 (KJ525928.1)	CN1	-
		17CW	C1 (KJ525912.1)	N1 (KJ525928.1)	CN1	I7 (KJ525942.1)
		18CW	C1 (KJ525912.1)	N1 (KJ525928.1)	CN1	-
**WARKOCZ (WAR) / MIDDLE VISTULA RIVER**	**POLAND**	1W	C1 (KJ525912.1)	N1 (KJ525928.1)	CN1	I20 (KJ525955.1)
		2W	C6 (KJ525917.1)	N4 (KJ525931.1)	CN10	-
		3W	C1 (KJ525912.1)	N1 (KJ525928.1)	CN1	I1 (KJ525936.1)
		4W	C1 (KJ525912.1)	N1 (KJ525928.1)	CN1	-
		5W	C1 (KJ525912.1)	N1 (KJ525928.1)	CN1	I21 (KJ525956.1)
		10W	C1 (KJ525912.1)	N1 (KJ525928.1)	CN1	-
		11W	C1 (KJ525912.1)	N1 (KJ525928.1)	CN1	I22 (KJ525957.1)
		13W	C1 (KJ525912.1)	N1 (KJ525928.1)	CN1	I20 (KJ525955.1)
		15W	C6 (KJ525917.1)	N4 (KJ525931.1)	CN10	I20 (KJ525955.1)

		17W	-	-	-	I10 (KJ525945.1)
		18W	C1 (KJ525912.1)	N1 (KJ525928.1)	CN1	I4 (KJ525939.1)
		19W	C6 (KJ525917.1)	N4 (KJ525931.1)	CN10	I23 (KJ525958.1)
CEDRON (CED) / UPPER ISTULA RIVER	POLAND	1C	C1 (KJ525912.1)	N2 (KJ525929.1)	CN2	I1 (KJ525936.1)
		5C	C1 (KJ525912.1)	N1 (KJ525928.1)	CN1	I2 (KJ525937.1)
		6C	C1 (KJ525912.1)	N3 (KJ525930.1)	CN3	I3 (KJ525938.1)
		8C	C1 (KJ525912.1)	N1 (KJ525928.1)	CN1	I4 (KJ525939.1)
		10C	C1 (KJ525912.1)	N1 (KJ525928.1)	CN1	I4 (KJ525939.1)
		11C	C1 (KJ525912.1)	N1 (KJ525928.1)	CN1	-
		12C	C1 (KJ525912.1)	N1 (KJ525928.1)	CN1	-
		15C	C1 (KJ525912.1)	N1 (KJ525928.1)	CN1	-
		16C	C1 (KJ525912.1)	N1 (KJ525928.1)	CN1	-
		23C	C1 (KJ525912.1)	N1 (KJ525928.1)	CN1	I5 (KJ525940.1)
		25C	C1 (KJ525912.1)	N1 (KJ525928.1)	CN1	-
		28C	C1 (KJ525912.1)	N1 (KJ525928.1)	CN1	-
SKAWINKA (SKA) / UPPER ISTULA RIVER	POLAND	1S	C1 (KJ525912.1)	N1 (KJ525928.1)	CN1	I16 (KJ525951.1)
		4S	C1 (KJ525912.1)	N1 (KJ525928.1)	CN1	I4 (KJ525939.1)
		6S	C1 (KJ525912.1)	N1 (KJ525928.1)	CN1	I17 (KJ525952.1)
		7S	C1 (KJ525912.1)	N1 (KJ525928.1)	CN1	-
		8S	C1 (KJ525912.1)	N1 (KJ525928.1)	CN1	-
		9S	C1 (KJ525912.1)	N1 (KJ525928.1)	CN1	-
		10S	-	N1 (KJ525928.1)	-	I18 (KJ525953.1)
		13S	C1 (KJ525912.1)	N1 (KJ525928.1)	CN1	I19 (KJ525954.1)
		22S	C1 (KJ525912.1)	N1 (KJ525928.1)	CN1	-
JASIOŁKA (JAS) / UPPER ISTULA RIVER	POLAND	1J	C1 (KJ525912.1)	-	-	
		3J	C1 (KJ525912.1)	N1 (KJ525928.1)	CN1	-
		7J	C1 (KJ525912.1)	N1 (KJ525928.1)	CN1	-
		8J	C1 (KJ525912.1)	-	-	-
		9J	C1 (KJ525912.1)	N1 (KJ525928.1)	CN1	-
		10J	C1 (KJ525912.1)	N1 (KJ525928.1)	CN1	I9 (KJ525944.1)
		14J	C1 (KJ525912.1)	N1 (KJ525928.1)	CN1	I10 (KJ525945.1)
		16J	C3 (KJ525914.1)	N1 (KJ525928.1)	CN5	I4 (KJ525939.1)
		18J	C1 (KJ525912.1)	N1 (KJ525928.1)	CN1	I11 (KJ525946.1)

		20J	C1 (KJ525912.1)	N1 (KJ525928.1)	CN1	I12 (KJ525947.1)
		23J	C3 (KJ525914.1)	N1 (KJ525928.1)	CN5	I10 (KJ525945.1)
		25J	C1 (KJ525912.1)	N1 (KJ525928.1)	CN1	I10 (KJ525945.1)
BABRUNGAS (BAB) / VENTA	LITHUANIA	1B	C7 (KJ525918.1)	N7 (KJ525934.1)	CN11	I24 (KJ525959.1)
		3B	C8 (KJ525919.1)	N8 (KJ525935.1)	CN12	-
		4B	C8 (KJ525919.1)	N8 (KJ525935.1)	CN12	-
		5B	C8 (KJ525919.1)	N8 (KJ525935.1)	CN12	I7 (KJ525942.1)
		6B	C9 (KJ525920.1)	N4 (KJ525931.1)	CN13	I7 (KJ525942.1)
		7B	C8 (KJ525919.1)	N4 (KJ525931.1)	CN14	-
		8B	C4 (KJ525915.1)	N4 (KJ525931.1)	CN6	-
		U5	C4 (KJ525915.1)	N4 (KJ525931.1)	CN6	I7 (KJ525942.1)
DUBYSA (DUB) / NEMAN	LITHUANIA	1D	C10 (KJ525921.1)	N4 (KJ525931.1)	CN15	I25 (KJ525960.1)
		2D	-	N4 (KJ525931.1)	-	-
		3D	C11 (KJ525922.1)	N8 (KJ525935.1)	CN16	I7 (KJ525942.1)
LUKNELIS (LUK) / NEMAN	LITHUANIA	1L	-	N8 (KJ525935.1)	-	I7 (KJ525942.1)
		2L	-	N4 (KJ525931.1)	-	I7 (KJ525942.1)
		3L	C4 (KJ525915.1)	N4 (KJ525931.1)	CN6	I20 (KJ525955.1)
		6L	-	N4 (KJ525931.1)	-	I26 (KJ525961.1)
		9L	-	N8 (KJ525935.1)	-	-
		10L	-	N8 (KJ525935.1)	-	-
		14L	-	N4 (KJ525931.1)	-	-
SESUVIS (SES) / NEMAN	LITHUANIA	9SE	C7 (KJ525918.1)	N7 (KJ525934.1)	CN11	-
		10SE	C8 (KJ525919.1)	N8 (KJ525935.1)	CN12	I7 (KJ525942.1)
		11SE	-	N4 (KJ525931.1)	-	I7 (KJ525942.1)
		12SE	C8 (KJ525919.1)	N8 (KJ525935.1)	CN12	I27 (KJ525962.1)
VIRVITA (VIR) / NEMAN	LITHUANIA	1V	C7 (KJ525918.1)	N7 (KJ525934.1)	CN11	I28 (KJ525963.1)
		2V	C7 (KJ525918.1)	N7 (KJ525934.1)	CN11	I29 (KJ525964.1)
		3V	-	N7 (KJ525934.1)	-	I4 (KJ525939.1)

		4V	-	N7 (KJ525934.1)	-	I7 (KJ525942.1)
		5V	C7 (KJ525918.1)	N7 (KJ525934.1)	CN11	-
**ZALVYS (ZAL) / NEMAN**	**LITHUANIA**	1Z	C8 (KJ525919.1)	N8 (KJ525935.1)	CN12	I4 (KJ525939.1)
		2Z	C7 (KJ525918.1)	N7 (KJ525934.1)	CN11	I11 (KJ525946.1)
		3Z	-	N7 (KJ525934.1)	-	-
		4Z	-	N4 (KJ525931.1)	-	-
		5Z	C8 (KJ525919.1)	N8 (KJ525935.1)	CN12	I7 (KJ525942.1)
		6Z	C7 (KJ525918.1)	N4 (KJ525931.1)	CN17	I4 (KJ525939.1)
		8Z	C8 (KJ525919.1)	N8 (KJ525935.1)	CN12	-
		10Z	-	N8 (KJ525935.1)	-	-

Table S2. Data on sampling localities from Poland and Lithuania

Geographical region	River	Locality code	Coordinates	Hydrological data	The conservation status of the species	Literature data
LITHUANIA	Babrungas	BAB	55° 56′ N<br>21° 53′ E	the right tributary of the Minija river, flows from the lake Plateliai, the catchment area about 270 km^2,	average abundance of *U. crassus* 21.8 ind./m2 (results of the *U. crassus* inventory carried in 2016)	www.upese.lt<br>Skujienė 2018
	Šešuvis	SES	55° 26′ N<br>22° 52′ E	the main tributary of the Jūra river, 115 km long, the catchment area about 1.916 km^2<br>there are almost no lakes in Šešuvis basin what caused its great seasonal fluctuations	Natura 2000<br>average abundance of *U. crassus* 1.2 ind./m2 (results of the first monitoring carried in 2008)	Skujienė 2018
	Zalvys	ZAL	55° 49′ N<br>25° 53′ E	the tributary of the Zalvė lake, flows from Duburis lake	Natura 2000<br>average abundance of *U. crassus* 10.5 ind./m2 (results of the first monitoring carried in 2008)	Skujienė 2018
	Virvita	VIR	55° 57′ N<br>22° 30′ E	the left tributary of the Venta river	average abundance of *U. crassus* 18.85 ind./m2 (results of the *U. crassus* inventory carried in 2016)	Skujienė 2018
	Dubysa	DUB	55° 57′ N<br>23° 4′ E	the tributary of Nemunas<br>Dubysa is connected with the Venta river by the abandoned Windawski Canal	Dubysa Regional Park established in 1992<br>*U. crassus* was found in state monitoring in 2014-2015	Skujienė 2018<br>Zettler et al. 2005
	Luknelis	LUK	55° 12′ N<br>25° 53′ E	15 km long, the right tributary of the Žeimenos river	detailed study along the river confirmed that *U. crassus* survived in Luknelė	www.upese.lt<br>Skujienė 2018

CENTRAL POLAND	TRIBUTARY	Pilica	PIL	50° 89′ N<br>19° 80′ E	the left-hand tributary of the Vistula River, flows into the middle Vistula<br>virtually isolated from the rest of the rivers of the Carpathian foothills but connected with Nida River by the ecological corridor - Biala Nida River	Natura 2000<br>Pilica River as well as its tributaries are the appropriate habitat for the thick shell river mussels	Abraszewska-Kowalczyk 2002; http://natura2000.org.pl/
		Czarna Włoszczowska	CZW	50° 95′ N<br>19° 85′ E	tributary of the Pilica River<br>a small geographical distance from Warkocz, but hydrologically isolated population	Natura 2000<br>low density of population, even though the conservation status of the species was determined as poor	Abraszewska-Kowalczyk 2002; http://natura2000.org.pl/
		Warkocz	WAR	50° 83′ N<br>20° 75′ E	tributary of the Lubrzanka River, than Czarna Nida River, Nida River and flows into the upper Vistula river<br>the residual remaining of large Nida population from the 70s; isolation from the 80s ; a small geographical distance from Czarna Włoszczowska, but hydrologically isolated population	Natura 2000<br>not very large, but stable, sampled individuals of different age classes, although, the conservation status of the species determined as unsatisfactory - the risks resulting from the anthropogenic influence	Piechocki 1981; http://natura2000.org.pl/
SOUTHERN POLAND	TRIBUTARY	Cedron	CED	49° 88′ N<br>19° 73′ E	tributary of the Skawinka River<br>hydrologically isolated population	Natura 2000<br>very large population; density 5-50 individuals/m^2 (dominated by juveniles)	Hus *et al*. 2006; http://natura2000.org.pl/
		Skawinka	SKA	49° 90′ N<br>19° 83′ E	the right-hand tributary of the upper Vistula River<br>once large, now rapidly declining population, isolated from remaining populations by the contaminated Vistula River; isolation from the 60s (personal communication, Zając)	Natura 2000<br>ow density of population, abnormal age structure; the conservation status of the species was determined as unsatisfactory	http://natura2000.org.pl/
		Jasiołka	JAS	49° 70′ N<br>21°67′ E	tributary of the Wisloka River which flows into the upper Vistula<br>isolated from remaining populations (personal communication, Zając)	Natura 2000<br>large and stable population (personal communication, Zając K.)	Hus 2003; http://natura2000.org.pl/
